# Cultivating physician empathy: a person-centered study based in self-determination theory

**DOI:** 10.1080/10872981.2024.2335739

**Published:** 2024-04-03

**Authors:** Adam Neufeld, Greg Malin

**Affiliations:** a Cumming School of Medicine, Department of Family Medicine, University of Calgary, Calgary, Alberta, Canada; b College of Medicine, Department of Academic Family Medicine, University of Saskatchewan, Saskatoon, Saskatchewan, Canada

**Keywords:** Physician, empathy, learning environment, basic psychological needs, self-determination

## Abstract

While physician empathy is a vital ingredient in both physician wellness and quality of patient care, consensus on its origins, and how to cultivate it, is still lacking. The present study examines this issue in a new and innovative way, through the lens of self-determination theory. Using survey methodology, we collected data from *N* = 177 (44%) students at a Canadian medical school. We then used a person-centered approach (cluster analysis) to identify medical student profiles of self-determination (based on trait autonomy and perceived competence in learning) and how the learning environment impacted empathy for those in each profile. When the learning environment was more autonomy-supportive, students experienced higher satisfaction and lower frustration of their basic psychological needs in medical school, as well as greater empathy towards patients. The translation into increased empathy, however, was only evident among the students with higher self-determination at baseline. Results from this study suggest that autonomy-supportive learning environments will generally support medical students’ psychological needs for optimal motivation and well-being, but whether or not they lead to empathy towards patients will depend on individual differences in self-determination. Findings and their implications are discussed in terms of developing theory-driven approaches to cultivating empathy in medical education.

## Introduction

Physician empathy is critical to quality patient care, and yet, how to instill it in medicine is an ongoing debate [1]. There are also unanswered questions in the literature about how empathy changes throughout medical training, and the potential negative implications for physicians’ psychological health [2]. Researchers have therefore called for interventions that target the curriculum – formal (e.g., increasing empathy training), informal (e.g., improving learning environments), and hidden (e.g., reducing student mistreatment) – to help cultivate physician empathy and wellness [1]. This call is well-founded; however, research thus far has neglected the role of motivational factors (both in the learning environment and in the person) and how they relate to physician empathy. Most quantitative studies have also used variable-centered and not person-centered approaches to analyses, which has limited our understanding of how to truly develop and support physician empathy. The present study draws on self-determination theory’s framework to address these gaps in the literature. It uses a person-centered approach to identify medical student profiles of self-determination and to explore how the learning environment shapes empathy towards patients for the students in each profile.

### Empathy in medical education

Empathy is broadly defined as understanding others’ emotional states and being able to express that understanding [3]. While consensus on how to specifically define empathy is missing, it is generally thought to be comprised of different facets – cognitive (identifying and comprehending a patient’s emotions and perspectives), emotional (imagining and sharing their psychological states), moral (being internally motivated to express empathy), and behavioural (communicating this understanding to the patient) [3]. Regardless of how it is conceptualized, there is agreement that more needs to be done to cultivate empathy in medical education [1]. Without it, patient satisfaction, patient engagement in medical decision-making, medical-legal risk, and clinical outcomes all suffer [4–6]. Though the reasons why have not been elucidated, empathy has also been linked to better physician mental health. In medical students, for example, higher empathy has been associated with better well-being, lower burnout, and higher ratings of clinical competence [7,8].

Despite this knowledge, how to cultivate and maintain physician empathy remains unclear. Ahrweiler et al. [2] noted the value of raising physician awareness about the psychosocial dimension of disease, as well as the influence of peers and role models, of managing personal stress and well-being, and of using reflective practice. Pohontsch et al. [9] further identified course structure (e.g., amount of emphasis on clinical empathy), students (e.g., life experience and maturity), patients (e.g., ‘easy’ and ‘difficult’ temperament), and conditions (e.g., the learning environment) as important factors. In other work, Dyrbye et al. [10] found that medical students who experienced mistreatment and perceived the learning environment less favourably were more apt to develop burnout, career regret, and display lower empathy than those with more positive experiences. Various types of interventions have also been shown to increase medical student empathy [11–15]. Most studies, however, have not been theory-driven and there are many inconsistencies and weaknesses in the current empathy intervention literature which limit our understanding of the phenomenon and how to support it [1].

### Self-determination theory and empathy

According to self-determination theory (SDT), satisfaction of basic psychological needs (autonomy, competence, and relatedness) is the motivational mechanism that energizes and directs people’s behaviour [16]. Autonomy is the need to feel that one’s behaviour is volitional (versus pressured or controlled); competence is the need to feel effective (versus incapable or a failure); and relatedness is the need to feel connected and cared for by others (versus disliked or excluded). SDT therefore considers environmental supports and barriers to meeting these needs, and autonomy in particular, as paramount in human motivation, development, and well-being [16].

Autonomy-supportive behaviours, themselves, are an act of empathy – e.g., when a physician takes their patients’ perspectives, acknowledges their feelings, provides them with a rationale, and offers them choices, in a non-judgmental and non-controlling manner. Individuals who receive autonomy support, such as patients from their doctors, clients from their therapists, or students from their instructors, are thus more likely to engage and persist in making behavioural change [17,18]. The reason for this is that autonomy support helps individuals meet their basic psychological needs, which promotes their autonomous motivation and perceived competence towards a specific task (e.g., striving to be more empathic towards others), even if the task is felt to be difficult or uninteresting. Prior research has demonstrated, for example, that when medical students sensed more instructor autonomy support and competence in their learning, they better internalized, integrated, and enacted the biopsychosocial values associated with patient-centered care, even 30 months later on [19]. Hence, creating autonomy-supportive learning environments represents a way to model and potentially instill empathy in aspiring physicians, which can support not only their self-determination and well-being, but the quality of their patient care.

While environmental factors like autonomy support are important to consider, dispositional factors like self-awareness (being aware of one’s feelings and sense of self) and perceived choice (feeling a sense of choices in determining one’s behaviour) are also relevant when it comes to physician wellness and quality of patient care. These factors reflect trait autonomy and are strong predictors of self-actualization, resistance to peer pressure, creativity, and empathy [20–22]. A physician’s capacity to be empathic towards their patients depends on their awareness of their own thoughts, feelings, and reactions, and how they influence patient care [23–25].

### A person-centered approach to studying empathy

A final consideration in what has limited our understanding of physician empathy (and thus how to promote it effectively) is that research tends to be ‘variable-centered’ in health professions education [26]. As Kusurkar et al. [27] point out, this research has its benefits but it can limit educators in knowing how to adapt their practices, since most studies focus on only a few variables and educational practices are complex and context dependent. ‘Person-centered’ research investigates how subgroups of individuals can be made within a sample (e.g., based on individual differences in certain factors), and how variable correlations differ for individuals in each subgroup or ‘profile’. This helps generate a more nuanced interpretation of data and improves our application of findings. Person-centered methods (e.g., cluster analysis) therefore tend to lend themselves better to understanding complex phenomena, and for working with students, compared to variable-centered methods. To date, there are no published person-centered studies concerning physician empathy, self-determination, and the learning environment, which are warranted given the intricate nature of empathy and our need to cultivate and sustain it in medicine.

### Current study

Guided by SDT, we investigated the relationship between the learning environment (i.e., instructor autonomy support), learners’ need satisfaction/frustration in medical school, and their empathy towards patients, and whether relations between these factors would vary based on students’ dispositional autonomy (self-awareness and perceived choice) and perceived competence in learning. We hypothesized that a more autonomy-supportive learning environment would bolster students’ psychological need fulfillment in medical school (higher satisfaction and lower frustration) and thereby facilitate their empathy; however, different profiles would exist based on individual differences in self-determination, which would influence to what extent.

## Methods

### Procedure

A total of 400 students from all four years of a Canadian medical program were invited to complete an online survey, running from April to July 2017. It contained demographic questions, including how students identified (male or female), current year of study (1, 2, 3, 4), and age (<22, 22–24, 25–27, 28–30, 30+), followed by scales related to self-determination and empathy (see *Measures*). All students were informed about the study and given a link to the survey tool, with two monthly reminders to participate. All gave informed consent. To protect confidentiality and minimize response bias, surveys were anonymous, and data was reported in aggregate form. This research received approval from the University of Saskatchewan Research Ethics Board (REB16–184).

### Measures

We used five commonly used scales, each with good reliability and validity evidence, to measure students’ perceived choice and self-awareness (i.e., trait autonomy) [21,28,29], perceived competence in learning the material in their medical school courses [19,30], the impact of the learning environment (i.e., instructor autonomy support) [19,31], aggregated need satisfaction and need frustration (i.e., autonomy, competence, and relatedness) in medical school [32–34], and empathy towards patients [35,36]. Each measurement instrument and their specifics are summarized in Table 1.

**Table 1.** Measurement instruments and scoring.

Instrument	Items	Measures	Scoring	Examples
Perceived Choice and Self-Awareness Scale (PCASS)	10	Self-awareness and perceived choice (i.e., dispositional autonomy)	1 (only A feels true) to 5 (only B feels true)	‘A. I always feel like it’s me choosing the things I do’ and ‘B. I sometimes feel that it’s not really me choosing the things I do’
Perceived Competence Scale (PCS)	10	Perceived competence in learning in one’s medical school courses	1 (not at all true) to 7 (very true)	‘I feel confident in my ability to learn the material in my courses’ and ‘I am able to achieve my goals in my courses’
Learning Climate Questionnaire (LCQ)	15	Perceptions of instructor autonomy support within the learning environment	1 (strongly disagree) to 7 (strongly agree)	‘I feel that my instructors provide me choices and options’ and ‘My instructors listen to how I would like to do things’
Basic Psychological Need Satisfaction and Frustration Scale (Work Domain)	24	Satisfaction and frustration of one’s autonomy, competence, and relatedness needs in medical school	1 (completely disagree) to 7 (completely agree)	‘I feel that my decisions in medical school reflect what I really want’ and ‘In medical school, I feel capable at what I do’ and ‘I feel that the people I care about in medical school also care about me’
Jefferson Scale of Physician Empathy – Medical Student Version (JSPE-S)	20	Clinical empathy	1 (strongly disagree) to 7 (strongly agree)	‘Patients feel better when their physician understands their feelings’ and ‘A physician’s sense of humour contributes to a better clinical outcome’

Note: For all self-report scales, except the JSPE-S, a mean score was calculated, where higher scores reflect higher levels of that construct. For the JSPE-S, a total mean score was calculated (ranging from 20–140), where higher scores reflect higher levels of clinical empathy.

### Data analysis

The software SurveyMonkey and SPSS version 26 were used for our survey and statistical analyses. Basic descriptive statistics were computed for the demographic and main study variables. We then computed Cronbach alpha reliability estimates for each scale and assessed how each of the study variables correlated, using Pearson correlation coefficients. All variables were checked for normality and linearity of relationships. Following these steps, we performed a *k*-means cluster analysis to identify distinct subgroups within the sample, using the three grouping variables: self-awareness, perceived choice, and perceived competence in learning in medical school. As mentioned, this approach allowed us to identify ‘hidden’ subgroups within the sample based on individual differences in motivational factors that, according to SDT, ought to play an integral role in shaping one’s need fulfillment, wellness, and empathy towards others.

To perform the clustering procedure, we first standardized all variables, then determined the optimal number of clusters by exploring a range of 0 to 10 clusters. We assessed the validity of results by assessing the cluster tendency, cluster iteration history, and analysis of variance (ANOVA) statistics. The stability of the cluster solution was assessed by carrying out a double-split cross-validation procedure [26]. We assessed whether the profiles differed by gender, year, and age, using chi-square tests, then compared each group in the variables of interest.

Finally, we tested our hypotheses through a series of simple and multiple regressions. These measured how: a) the learning environment related to students’ need satisfaction/frustration in medical school, and b) students’ need satisfaction/frustration related to their empathy. We examined effects of cluster via the ‘split file’ and ‘organize output by groups’ functions in SPSS.

## Results

### Participants

In total, 177 students (44%) completed the survey: 75 males (42%) and 102 females. There were 66 first years (37%), 42 second years (24%), 37 third years (21%), and 32 fourth years (18%). Most students (93.8%) were 22–30 years old. This was a representative sample based on the medical student population in Canada [37]. Note: some students did not complete some of the scales, so we carried out the analyses with the scores that were obtained.

### Variable relationships

As seen in Table 2, self-awareness, perceived choice, perceived competence in learning, and the autonomy-supportiveness of the learning environment, each positively related to need satisfaction and negatively related to need frustration in medical school. Self-awareness, perceived competence in learning, and need satisfaction, positively related to empathy. These findings were expected based on the SDT literature [20–22]. Results showed that there was no association between age or gender and any other variable. Year of study negatively correlated with perceived choice (*r* = −.16, *p* < .05). All scale reliabilities were considered satisfactory.

**Table 2.** Descriptives, correlations, and reliabilities for study variables.

	1	2	3	4	5	6	7
1. AWS	(.70)						
2. PCH	.39**	(.86)					
3. PCL	.43**	.26**	(.90)				
4. LC	.37**	.38**	.34**	(.93)			
5. BNS	.46**	.50**	.56**	.59**	(.88)		
6. BNF	−.52**	−.52**	−.55**	−.62**	−.75**	(.87)	
7. EMP	.16*	.07	.19*	.02	.26**	.08	(.77)
Mean	3.8	3.3	5.5	4.7	4.7	3.3	5.7
SD	0.9	0.9	1.1	1.0	0.9	1.0	0.5

AWS, awareness of self; PCH, perceived choice; PCL, perceived competence in learning; LC, learning climate; BNS, aggregated basic need satisfaction in medical school; BNF, aggregated basic need frustration in medical school; EMP, empathy. Cronbach alphas along the diagonal.

*p < .05, **p < .01.

### K-means cluster analysis

**Figure 1.**
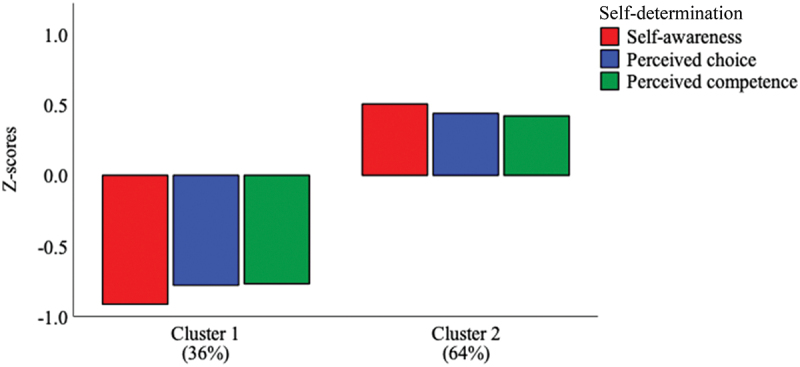
Bar plot of clusters and features by study variables.

We tried fitting various cluster solutions and results confirmed that a 2-factor solution best represented the data, with a change in cluster centers equal to .000 for both groups and complete convergence after 6 iterations. The corresponding ANOVA confirmed statistically significant differences between the two profiles (which we conceptualized as ‘low’ and ‘high’ in self-determination) across each factor – self-awareness (*F* (1, 164) = 139.31, *p* < .001), perceived choice (*F* (1, 164) = 84.32, *p* < .001), and perceived competence in learning (*F* (1, 164) = 78.75, *p* < .001). Each profile’s characteristics are depicted in Table 3 and Figure 1. Results of the double-split cross-validation assessment yielded very similar cluster solutions, thereby supporting the validity and stability of these results.

**Table 3.** Distribution of medical students by cluster.

Cluster membership	Cluster 1 ‘Low’ self-determination	Cluster 2 ‘High’ self-determination	Total
No. students per cluster (%)	59 (36%)	107 (64%)	166 (100%)
*Gender*			
Males	22 (37%)	50 (47%)	72 (43%)
Females	37 (63%)	57 (53%)	94 (57%)
Chi square statistic	1.38	-	-
Significance	*p* = .24	-	-
*Year*			
Year 1	18 (30%)	45 (42%)	63 (38%)
Year 2	20 (34%)	19 (18%)	39 (23%)
Year 3	13 (22%)	21 (20%)	34 (20%)
Year 4	8 (14%)	22 (20%)	30 (18%)
Chi square statistic	6.69	-	-
Significance	*p* = .08	-	-
*Age*			
< 22 years	0 (0%)	4 (4%)	4 (2%)
22–24 years	36 (61%)	58 (54%)	94 (57%)
25–27 years	14 (24%)	25 (23%)	39 (24%)
28–30 years	7 (12%)	12 (11%)	19 (12%)
>30 years	2 (3%)	8 (8%)	10 (6%)
Chi square statistic	4.39	-	-
Significance	*p =* .62	-	-

### Between-cluster differences

We next explored how students in each profile differed in their self-awareness, perceived choice, perceived competence in learning, and in particular, their perceptions of the learning environment, need satisfaction/frustration in medical school, and empathy towards patients. Levene’s test was checked for each factor and adjusted degrees of freedom were used wherever error variances were unequal between groups. There were no significant demographic differences between the two profiles (see Table 3). All further analyses thus did not control for these factors.

Based on these results, we proceeded with independent samples *t*-tests to compare the students in each profile. As seen in Table 4, the students in cluster 2 (with higher self-determination) perceived significantly more autonomy support within the learning environment, had higher need satisfaction and lower need frustration in medical school, and had more empathy towards patients, compared to the students in cluster 1 (with lower self-determination). The associated Cohen’s *d* effect sizes of profile were medium to large.

**Table 4.** Means, standard deviations, and *t*-test results by cluster.

	Cluster 1 (‘low’)	Cluster 2 (‘high’)	*t*-test for equality of means							
								*95% CI*		
	*M (SD)*	*M (SD)*	*t*	*df*	*Sig.*	*MD*	*SE*	Lower	Upper	*d*
AWS	3.04 (.83)	4.25 (.48)	11.80	80	.000	1.20	.10	−1.41	−1.00	1.78
PCH	2.57 (.78)	3.66 (.71)	9.18	164	.000	1.10	.12	−1.33	−.86	1.46
PCL	4.66 (1.04)	5.95 (.81)	8.28	97	.000	1.30	.16	−1.61	−.99	1.38
LC	4.04 (1.07)	5.12 (.77)	7.47	91	.000	1.08	.14	−1.37	−.80	1.16
BNS	3.99 (.80)	5.09 (.67)	9.29	163	.000	1.09	.12	−1.32	−.86	1.49
BNF	4.06 (.83)	2.79 (.81)	9.59	164	.000	1.27	.13	−1.01	1.53	1.55
EMP	5.56 (.48)	5.76 (.48)	2.45	164	.015	.19	.08	−.34	−.04	.41

AWS, awareness of self; PCH, perceived choice; PCL, perceived competence in learning; LC, learning climate; BNS, aggregated basic need satisfaction; BNF, aggregated basic need frustration; EMP, empathy; *M*, mean; *SD*, standard deviation; *df*, degrees of freedom; *Sig*., significance level (two-tailed); *MD*, mean difference; *SE*, standard error of mean difference; *CI*, confidence intervals; *d*, Cohen’s effect size.

### How self-determination impacts physician empathy

Following the subgroup comparisons, we ran a series of regressions (see *Statistical Analyses*). First, results showed that there was a positive association between the autonomy-supportiveness of the learning environment and students’ need satisfaction in medical school, both for those in cluster 1 (*β* = .58, *p* < .001, *CI* = .31 to .75) and cluster 2 (*β* = .41, *p* < .001, *CI* = .23 to .55). The learning environment accounted for 33.5% of the variance in need satisfaction for those in cluster 1 and 16.9% for those in cluster 2. Similarly, we found a negative association between the autonomy-supportiveness of the learning environment and students’ need frustration in medical school, again for those in cluster 1 (*β* = −.52, *p* < .001, *CI* = −.62 to -.21) and cluster 2 (*β* = −.50, *p* < .001, *CI* = −.66 to −.34). The learning environment accounted for 26.7% and 25.4% of the variance in need frustration for those in each cluster, respectively. A multiple regression then confirmed that need satisfaction (*β* = .44, *p* < .001, *CI* = .26 to .82) and need frustration (*β* = .29, *p* = .01, *CI* = .08 to .61) in medical school both predicted higher empathy towards patients, but only for the students in cluster 2 (*p* = .001), with an explained variance of 11.2%. For those in cluster 1, need satisfaction (*β* = .25, *p* = .21) and need frustration (*β* = .24, *p* = .24) in medical school did not relate to empathy towards patients (*p* = .41).

## Discussion

In the present study, we used a person-centered approach to explore the relationship between the learning environment and medical student empathy, and how differences in students’ self-awareness, perceived choice, and perceived competence in learning, played a role. The following section provides potential explanations for our results and what their implications are for teaching and learning in medicine and health professions education more broadly. A strengths and limitations section follows to help guide future research.

### Identification of profiles and their relations

This study identified two medical student profiles of self-determination. Medical students in the first profile displayed lower self-awareness, perceived choice, and perceived competence towards learning in their courses. Those in the second profile displayed higher self-awareness, perceived choice, and perceived competence in learning in their courses. While other combinations of profiles were (and are) plausible, the clustering results indicated that this 2-cluster solution best represented the sample. Interestingly, we observed that roughly 65% fell into the ‘high’ group compared to the ‘low’ group. This suggests that in a typical cohort of medical students, most will likely fall into either of these two ‘self-determination’ categories, and that about one third will perceive lower autonomy and competence.

With that in mind, we compared each group’s perceptions of the learning environment, need fulfillment in medical school, and empathy towards patients. As expected, they differed substantially and with medium to large effect. The more self-determined students perceived greater autonomy support, had higher need satisfaction and lower need frustration in medical school, and had higher empathy. These findings suggest that medical students who are more self-aware and feel more agentic and confident in their learning will be more attuned to their instructors’ motivational support, experience more need fulfillment in their medical education, and possess more empathy towards patients. Conversely, the opposite will be true for those who are less self-aware and feel more pressured and unconfident in their learning. These results shed new light on how medical learners will orient to the same environment, differently, and why those nuances matter with respect to their empathy – illustrating the ‘one size does not fit all’ principle [27]. While we did not measure well-being in this study, instructor autonomy support and need fulfillment have been strongly implicated in medical students’ well-being [31,38,39]. Hence, our findings may also help explain the associations in the literature between physician empathy and wellness.

### A person-centered model of physician empathy

Of note, when we first assessed how our variables correlated, there was no obvious link between the learning environment and students’ empathy towards patients. Other correlational studies have reported similar null findings [40]. Through subsequent person-centered analyses, however, this relationship crystalized. We observed that when the learning environment was more autonomy-supportive, it related to greater satisfaction and lower frustration of students’ autonomy, competence, and relatedness needs in medical school, and that this related to higher empathy. Importantly, though, this was only the case for the medical students with higher self-awareness, perceived choice, and perceived competence in their learning. Accounting for these otherwise hidden precursors helped to reduce the ‘noise’ in our data, allowing us to better appreciate the interaction between medical student, learning environment, and empathy towards patients. These findings closely align with Laughley et al.’s [41] garden model of empathy – beginning with the innate seeds of empathy that students bring to medical school (i.e., autonomy and competence in the person) and the flowering of empathy as a fragile process, subject to enablers and disablers within the learning environment (i.e., autonomy support versus control by instructors).

One finding that stood out within the ‘high’ group (cluster 2) was the positive association between their need frustration in medical school and empathy. The direction of this relationship was unexpected and implies that students with more self-awareness, perceived choice, and confidence in their learning, will convert both need-satisfying *and* need-frustrating experiences into expressions of empathy towards patients. This might be because individuals with higher autonomy tend to face and grapple with (versus avoid or suppress) their negative emotions and experience more resilience and well-being as a result [42]. Either way, this particular finding helps explain why the learning environment might hinder some medical students’ empathy and wellness but not others’ [10,40], and how we might support those students from a self-determination perspective. Practical examples of how we might go about doing this are described below.

### Theoretical & practical implications

This study aimed to advance our understanding of how to cultivate physician empathy, in a person-centered way. We outline this because our findings identify groups of students who may struggle more than others, psychologically and empathically, and it is critical that we use results constructively, to not stigmatize them. In other words, results are not meant to imply that certain individuals ought to be targeted or taught differently. They simply demonstrate why a person-centered and theory-driven approach is useful for understanding the complex nature of empathy, and how to foster it. In line with SDT, our results show that providing autonomy-supportive learning environments is beneficial for all medical students but that to promote empathy towards patients, we can place more emphasis on particular individual aspects (i.e., awareness, choices, and confidence) that will benefit, to a greater extent, those with lower self-determination. The person-centered findings also highlight why creating a blanket-style approach to teaching empathy in medical education would have limited impact for various students.

Examples of how to emphasize these motivational factors could be in tailoring curricula: formal (what to teach with empathy), informal (how to improve learning environments), and hidden (addressing factors that overwhelm students, such as workload) [2,31]. Our findings may also help guide interventions that cultivate medical student empathy through autonomy-supportive SDT principles – for instance, in workshops for medical teachers on how to support medical learners’ basic psychological needs [31], and sessions for medical students on how to support patient autonomy, coupled with patient feedback [43]. Regardless of whether a medical student is ‘high’ or ‘low’ in self-determination, we would do well to create psychological safety for them, foster their self-awareness, and help them recognize choices in when and how to be empathic with patients.

Supporting self-awareness and perceived choice could simply be encouraging medical learners to: a) keep an open mind, b) consider how their actions affect others, c) stay focused and self-reflect (e.g., before, during, and after patient encounters), d) contemplate their strengths and weaknesses, e) embrace their intuition (e.g., with how to navigate and adjust during difficult patient encounters), f) know their emotional triggers, g) set boundaries, and h) practice self-discipline [44]. All of these facets relate to mindfulness, which we know is an important facilitator of medical learners’ need satisfaction, healthy coping, and wellness [39]. That need frustration positively related to empathy for most in this study suggests that helping students process and integrate their negative experiences in medical school would also be of value [45]. The key is doing things in an autonomy-supportive way, based on the unique goals and challenges that medical students face at each stage of their medical training (e.g., desiring more choices in their senior years).

### Strengths & limitations

This study has several limitations which may guide future research. First, the data was from self-report surveys collected at a single institution. As others have highlighted, this may limit generalizability in that self-report measures may not translate to patient-report or behavioural outcomes when it comes to empathy [1]. Self-report measures of empathy may also be subject to social desirability bias, which makes it difficult to discern whether interventions truly increase empathy or just physicians’ awareness of its importance. We chose to use the Jefferson Scale of Physician Empathy (JSPE) because it is the most used scale in the literature [1], and it has been validated against behavioural and patient-report measures [46], with the strongest correlations among real patients [36]. Future studies would nonetheless do well to adopt a multi-informant approach, including both physicians and patients.

Findings are also correlational and based on a cross section of only medical students (and not residents, fellows, or attending physicians). This too may limit generalizability and it prevents causal conclusions the way that experimental designs might permit. Like empathy, some constructs we measured are also subject to change over time and to vary between individuals at different educational stages. For example, empathy towards patients might change between medical school, residency, and clinical practice. We would therefore recommend adapting this study to measure cohort effects, since this would further our knowledge, not only on *how* but *why* physician empathy might change throughout medical training [2]. We attempted to mitigate these limitations by using a person-centered approach, well-established measurement instruments (which showed strong internal consistency in this study), and by grounding our hypotheses in SDT’s well-supported motivation and well-being framework. These aspects strengthen the reliability, convergent validity, and applicability of our findings.

## Conclusions

Guided by SDT, and using a person-centered approach, the present study identified two profiles of self-determination among medical students (based on levels of trait autonomy and perceived competence in learning) and illustrated how – through providing autonomy-supportive learning environments and fostering students’ basic psychological needs in medical school – we can model and help them develop their empathy. Findings offer new insights, not only on why some medical students – and by extension, physicians – may demonstrate more empathy towards patients than others, but also how medical educators can play a role in shaping physician empathy, in an evidence-based way.
